# Efficacy of a pressure-sensing mattress cover system for reducing interface pressure: study protocol for a randomized controlled trial

**DOI:** 10.1186/s13063-015-0949-x

**Published:** 2015-09-29

**Authors:** Holly Wong, Jaime Kaufman, Barry Baylis, John M. Conly, David B. Hogan, Henry T. Stelfox, Danielle A. Southern, William A. Ghali, Chester H. Ho

**Affiliations:** W21C Research and Innovation Centre, Cumming School of Medicine, GD01 Teaching Research & Wellness Building, University of Calgary, 3280 Hospital Drive, NW, Calgary, AB T2N-4Z6 Canada; Division of General Internal Medicine, Cumming School of Medicine, University of Calgary, Calgary, AB Canada; Infection Prevention and Control, Alberta Health Services, Calgary, AB Canada; O’Brien Institute for Public Health, University of Calgary, Calgary, AB Canada; Department of Medicine, Cumming School of Medicine, University of Calgary, Calgary, AB Canada; Snyder Institute for Chronic Diseases, Cumming School of Medicine, University of Calgary, Calgary, AB Canada; Brenda Strafford Foundation (Geriatric Medicine), University of Calgary, HSC-3330 Hospital Dr. NW, Calgary, AB T2N 4N1 Canada; Department of Critical Care Medicine, Cumming School of Medicine, University of Calgary, Calgary, AB Canada; Department of Community Health Sciences, Cumming School of Medicine, University of Calgary, Calgary, AB Canada; Alberta Health Services, Alberta, Canada; Division of Physical Medicine & Rehabilitation, Department of Clinical Neurosciences, Foothills Hospital, University of Calgary, 1403 - 29th Street NW, Calgary, AB T2N 2T9 Canada; Foothills Medical Centre, Special Services Building, Ground Floor, AGW5, Calgary, AB T2N 2T9 Canada

**Keywords:** pressure ulcers, decubitus ulcers, bedsores, continuous pressure mapping, interface pressure imaging

## Abstract

**Background:**

Interface pressure is a key risk factor in the development of pressure ulcers. Visual feedback of continuous interface pressure between the body and support surface could inform clinicians on repositioning strategies and play a key role in an overall strategy for the prevention and management of pressure ulcers.

**Methods/Design:**

A parallel two-group randomized controlled clinical trial will be conducted to study the effect of continuous pressure imaging on reducing interface pressure and on the incidence of pressure ulcers in vulnerable hospital patients. A total of 678 eligible consenting inpatients at risk of pressure ulcer development in a tertiary acute care institution will be randomly allocated to either having the ForeSite PT™ system with the liquid-crystal display monitor turned on to provide visual feedback to the clinicians while also collecting continuous interface pressure data (intervention group) or to having the ForeSite PT™ system with monitor turned off (that is, not providing visual feedback) but still collecting continuous interface pressure data (control group), in a ratio of 1:1. Continuous interface pressure data will be collected in both groups for 3 days (72 h). Data collection will continue until discharge for a subset of approximately 60 patients. The primary outcome will be the differences in the two groups’ interface pressure analysis. Interface pressure readings will be collected through hourly samplings of continuous interface pressure recordings. Secondary outcomes will be the differences between the two groups in pressure-related skin and soft tissue changes in areas at risk of pressure ulcer (obtained at baseline within 24 h of admission) and on the third day of the trial or at discharge and perceptions of the intervention by patients and clinicians (obtained on the third day or at discharge).

**Discussion:**

This will be the first randomized controlled trial to investigate the effect of visual feedback with continuous interface pressure of vulnerable hospital patients across different care settings, and the association between interface pressure and development of pressure-related skin and soft tissue changes. The results could provide important information to guide clinical practice in the prevention and management of pressure ulcers.

**Trials registration:**

ClinicalTrials.gov NCT02325388 (date of registration: 24 December 2014).

## Background

Pressure ulcers are a leading cause of morbidity for in-facility individuals [1] and lead to substantial discomfort, prolonged hospitalizations, additional costs, and in some cases death [2]. A pressure ulcer is defined as “a localized injury to the skin and/or underlying tissue over a bony prominence, as a result of pressure, or pressure in combination with shear” [3]. Their severity varies from skin erythema to full thickness tissue loss with damage extending into muscle and bone [3]. Annually, pressure ulcers affect an estimated 250,000 to 500,000 individuals in Canada with an overall estimated prevalence of 26.0 % in healthcare institutions [1]. In Germany, the prevalence rate is estimated to be 10 to 25 % among ward patients and as high as 30 % in rehabilitation centers [4]. In one Austrian public hospital, incidence rates were between 1.39 % and 7.98 % for Stage 1, 0.14 % and 1.52 % for Stage II, and 0 % and 0.88 % for Stage III pressure ulcers [5].

More severe cases require intensive therapy, have longer healing times and are associated with a higher incidence of complications [6]. In the United States, the average hospital treatment cost associated with stage IV pressure ulcers range from $124,327 to $129,248 USD [7], whereas in the UK the estimated cost of treatment varies from £1,214 to £14,108 per case [6]. Similar costs are seen in Canada, where the estimated average monthly cost of pressure ulcer management among individuals with a spinal cord injury in 2010 was $4,475 CDN [8].

The development of pressure ulcers is multifactorial. General risk factors for their development include limited mobility and activity causing excessive interface pressure between the skin and the supporting surface; compromised tissue perfusion (associated with conditions such as diabetes and anemia); a variety of skin conditions; excessive skin moisture (for example, from urinary incontinence); advanced age; malnutrition; and poor general health status [9–11]. Additional predictors of pressure ulceration among adults in the intensive care unit (ICU) include admission duration, norepinephrine administration and, the presence of cardiovascular disease [12]. Among adults with spinal cord injuries, delayed and/or inappropriate medical management, history of or existing pressure ulceration and limited independence in self-care are predictive of their development [13].

Among at-risk individuals, pressure ulcers develop on body areas exposed to unrelieved high interface pressure over prolonged periods of time [14, 15]. If interface pressure is higher than mean capillary blood pressure, blood flow can be compromised, with the affected areas becoming ischemic and necrotic if the duration of pressure is sufficiently sustained [14]. Though commonly accepted that an area becomes at-risk when interface pressure exceeds 30 to 32 mmHg [16–20], it must be emphasized that the duration of the interface pressure is equally as important as the magnitude of the interface pressure.

As pressure applied over a prolonged period is a key risk factor in the development of pressure ulcers, frequent and regular repositioning of the patient is a common practice that has been recognized for decades as an integral component of prevention and treatment protocols [15]. To date, the published literature has established neither repositioning nor the frequency of repositioning to be effective at preventing and managing pressure ulceration. One Cochrane systematic review concluded that there is limited robust evidence on the effectiveness of positioning and frequency of repositioning for pressure ulcer prevention [21]. Similarly, another Cochrane review could not find any randomized trials that assess the effectiveness of repositioning on healing rates of pressure ulcers [22]. Thus while repositioning is rational and widely recommended, the lack of robust evaluations of how repositioning alters interface pressure has led to uncertainty and a clear need for high-quality, adequately powered trials to assess different approaches to the implementation of repositioning [21]. It is generally accepted that many pressure ulcers are avoidable though there are also situations where they cannot be prevented [23]. Due to the complexity of pressure ulcer development, many acute and long-term care facilities adopt multipronged, multidisciplinary interventions to prevent pressure ulcers [24]. Current best practice guidelines recommend a standardized pressure ulcer risk assessment [25]. The Braden Scale for Predicting Pressure Sore Risk [26] (herein the “Braden Scale”) is the most validated with good sensitivity/specificity balance and predictive abilities [27]. Nevertheless, these strategies still do not directly address the relation between repositioning and interface pressure.

Since inadequate pressure redistribution is recognized as a major risk factor for the development of pressure ulcers, continuous pressure imaging (“CPI”) of the interface pressure between the body and support surface could play a role in an overall preventive strategy for pressure ulcers. A thin mattress cover with embedded interface pressure sensors and continuous monitoring capabilities could provide real-time interface pressure measurements over anatomical pressure points in a format viewable to healthcare providers, patients and their families on a bedside liquid-crystal display (“LCD”) monitor. Caregivers and/or healthcare providers could utilize these data to determine when and how a patient should be repositioned in order to redistribute optimally the interface pressure in real time [17, 28–30]. A recent prospective controlled study indicated that CPI could decrease the incidence of pressure ulcers in a medical intensive care unit [31]. In this study, only 0.9 % of patients with the intervention developed pressure ulcers graded at Stage II, as compared to 4.8 % of the patients in the control group who developed them, also at the Stage II level. In a another study over a 2-month period, only one patient (0.03 %) in the group of patients placed on beds with a continuous bedside pressure mapping (CBPM) system developed a pressure ulcer, whereas 16 patients (5 %) of the historical control group of patients placed on the same beds without CBPM (*P* = 0.001) [32]. Further research is required to assess whether this technology can have similar positive effects in other care settings [28].

This study will use XSENSOR Technology Corporation’s ForeSite PT™ Patient Turn System (referred to as the “ForeSite PT™ system”), a patient repositioning reminder system utilizing CPI technology through a pressure-sensing mattress cover, to test the efficacy of this approach in reducing interface pressure among at-risk hospitalized patients. Our team has previously undertaken an evaluation of the ForeSite PT™ system, demonstrating a positive effect of the technology on purposeful turning of patients [33]. However, the goal of this prior study was not to measure changes in actual interface pressure. This proposed study will build on our previous XSENSOR research to test the efficacy of CPI in reducing interface pressure for at-risk patients. As the most recent Canadian publication on estimates of pressure ulcer occurrence is relatively outdated [1], this study will provide updated data as well as help inform the Canadian context.

The primary study objective is to test the efficacy of a CPI system (ForeSite PT™ system) in its ability to reduce interface pressure as reflected by a composite interface pressure analysis.

Secondary objectives include the following:

1. to assess whether CPI reduces the incidence of pressure-related tissue changes,
2. to determine the minimum interface pressure associated with higher risk for pressure ulcer development, and
3. to assess healthcare provider and patient perceptions of this CPI system.

Specifically, the study will address the following research questions:

1. Is CPI efficacious in reducing interface pressure by assisting healthcare providers in determining the need and type of patient turning/repositioning that would result in lower interface pressure?
2. Does the application of CPI reduce the incidence of pressure-related skin changes?
3. Does interface pressure correlate with clinical outcomes?
4. What are the perceptions (both positive and negative) of healthcare providers to CPI with regard to the functionality, ease of use and interpretation of pressure data on the monitor?
5. What are the perceptions (both positive and negative) of patients (and if appropriate, family members) to CPI (including mattress cover and monitor display of their pressure points)?

## Methods/Design

Ethics approval for this study was obtained from the Conjoint Health Research Ethics Board of the University of Calgary (REB13-0794).

### Trial design

The trial will take place in a tertiary acute care center situated in a major urban center. Participants will be recruited from nursing units that care for patients with a high risk of pressure ulcer development, including acute medical, neurology, neurosurgery and intensive care units. A parallel, two-group, randomized controlled design will be utilized to assess the effect of CPI on interface pressure, pressure-related skin and soft tissue changes and the perceptions of healthcare providers and patients. Eligible consenting inpatients will be randomly allocated to either one of two groups in a ratio of 1:1 and then followed for 3 days (72 h) or until discharge. The intervention group will have the ForeSite PT™ system and the LCD monitor turned on to provide visual feedback to the healthcare providers while collecting continuous interface pressure data. The control group will have the ForeSite PT™ system turned on for collecting continuous interface pressure data, but the LCD monitor will be turned off so as not to provide visual feedback to the healthcare providers. As a safeguard against use of the monitor for visual feedback among the control group, the monitor’s brightness controls will be adjusted to limit visibility and a cover will be placed over the monitor. Interface pressure readings will be analyzed using hourly snapshot samples of continuous interface pressure recordings. Clinical outcomes will be obtained at baseline (within 24 h of admission) and on the third day of the trial. The perceptions of patients allocated to the intervention group and the perceptions of healthcare providers each time a patient in their care is allocated to the intervention group will be measured on the third day. A subset of 60 participants from the study (30 intervention, 30 control) will have data collection until their discharge from the hospital in order to allow for a longer duration for clinical outcomes to be evaluated.

### Participants

#### Recruitment

The charge nurse, and/or the patient care manager, and/or clinicians on their respective units, will facilitate the identification of eligible inpatients by a daily review of new admissions. After an eligible patient has been identified, the research nurse or the research assistant will ask him or her (or a proxy decision-maker, if not capable of consenting), if he or she would be interested in participating in a study to evaluate a pressure sensing technology that may provide real-time feedback to healthcare providers. If the patient agrees to participate, he or she will be asked to provide written informed consent prior to being enrolled in the study. Once the patient provides written informed consent, the research nurse will set up the CPI system, including placing the sensor mattress cover under the linen and mounting the LCD monitor to the headwall. The research nurse will ensure that the sensor mattress cover and monitor are functioning properly on a daily basis and will instruct housekeeping on sterilization practices of the device components.

#### Eligibility

The selection of patients will be based on the following inclusion and exclusion criteria.

The inclusion criteria are as follows:

1. Adult males or females, with a minimum age of 18 years old.
2. Expected to have a length of stay on the unit of at least 3 days.
3. Require assistance with bed mobility or completely dependent for bed mobility as determined by the “Bed” components in the de Morton Mobility Index (DEMMI).
  Eligible patients would meet the following criteria:
  - Unable (score of “0”) to bridge, roll onto their side, and sit from lying supine.
  - Unable (score of “0”) to bridge and roll onto their side, and requires minimal
  - Able (score of “1”) to bridge, unable to roll onto their side (score of “0”), and requires minimal assistance and/or supervision with sitting from lying supine.
4. Capacity to provide consent, or have a surrogate decision-maker to provide consent on their behalf.
5. Not near the end of life within 3 days of enrollment in the study.

The exclusion criteria are as follows:

1. Have a planned admission to another unit (including those identified as a setting for data collection in the study) within three days of enrollment in the study.
2. Sleep in a chair at night.
3. Any patients whose clinical care would be negatively impacted if turned or repositioned.

#### Setting

This trial will take place in at least five inpatient nursing units at the Foothills Medical Centre, a tertiary care institution, in Calgary, Alberta, Canada. The five units include, but are not limited to the following: 1) Unit 36 and Unit 37 in the Special Services Building, the internal medicine services that care for vulnerable general medical inpatients with immobility arising from complex multisystem medical illness; 2) the intensive care unit (“ICU”) that cares for sedated and immobilized patients requiring life support for prolonged periods of time; and 3) Unit 101 and Unit 112, acute neurological units that care for patients with mobility impairment resulting from neurological illnesses, spinal cord and/or significant head injuries. These units were chosen due to their high concentration of patients at high-risk of pressure ulcer development and the feasibility of data collection, as demonstrated by a pilot study that was performed by our group in preparation for this RCT.

### Intervention

All eligible consenting inpatients will receive usual care by their healthcare providers and undergo continuous interface pressure monitoring by the ForeSite PT™ system throughout all 3 days of enrollment or until discharge. Three days was chosen as the duration for four reasons: 1) it is the average length of stay in the inpatient units where this trial is taking place, and extending the enrollment period beyond 3 days when patients may be transferred to another nursing unit may introduce more variability in the nursing practice and care and would not be feasible if they are discharged to another facility or home; 2) expert consensus of the investigation team deemed that 3 days was sufficient for pressure-related skin and soft tissue changes to develop; 3) there is likely to be change in nursing shifts and personnel beyond 3 days, thereby introducing more variables; and 4) as interface pressure will be continually collected throughout the enrollment period, a longer duration would result in an extremely large volume of data. Thus, 3 days will allow for optimal data collection while maintaining feasibility of participant enrolment.

Following the 3-day enrollment period, a subset of 60 participants who have not yet been discharged will continue to undergo continuous interface pressure monitoring until they are discharged from the hospital. This subset of participants will enrich the dataset by providing a longitudinal perspective that can inform a potential larger multicenter RCT.

**Fig. 1:**
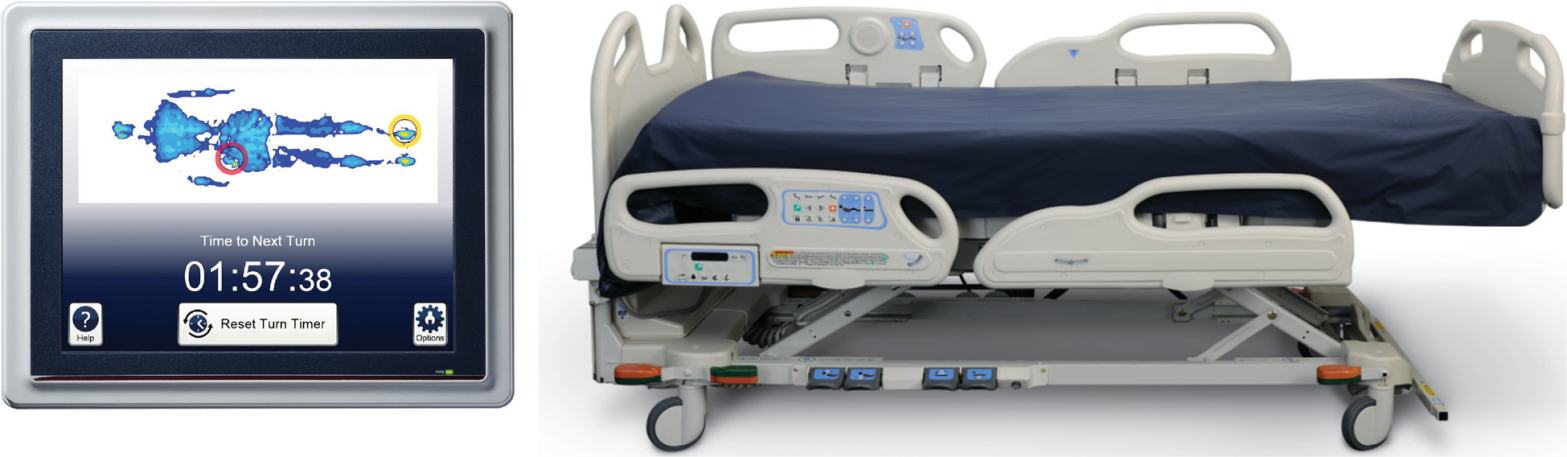
ForeSite PT™ Patient Turn System

XSENSOR Technology Corporation’s ForeSite PT™ Patient Turn System (referred to as the “ForeSite PT™ system”) continuously monitors the interface pressure and provides CPI that quantifies real-time interface pressure information (Fig. 1). It also provides patient turn tracking to assist with management of the patient turn schedule by alerting healthcare providers and/or caregivers to the location of body areas that have experienced the greatest exposure and when the next turn/repositioning is due as per pre-set alerts.

The ForeSite PT™ system consists of two parts: a thin, flexible sensor mattress cover that is positioned under the hospital linen (that is, fitted mattress sheet) and an LCD monitor that is mounted to the head of the bed. The LCD monitor displays color information about the patient surface pressure on the bed and provides risk information determined by the length of time pressure had persisted in any location.

**Fig. 2:**
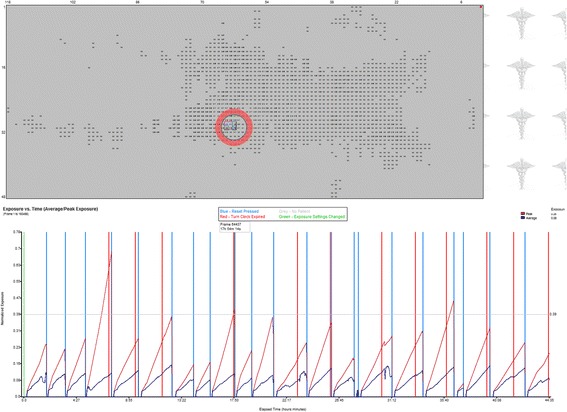
A pressure imaging session processed by XSENSOR Pressure Exposure Analyzer Tool (PEAT)

**Fig. 3:**
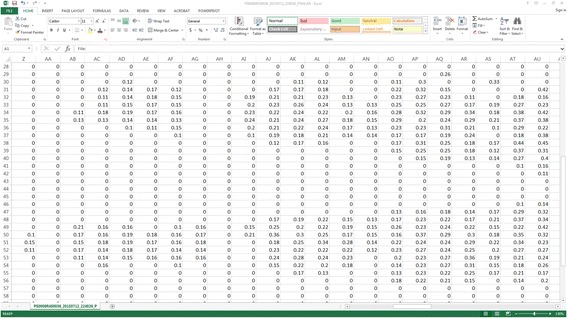
Processed pressure imaging session extracted in CSV format

The measurements for interface pressure will be achieved by the continuous collection of interface pressure readings by the ForeSite PT™ system’s pressure sensing mattress cover, which is a capacitive sensor array with a spatial resolution of 0.625. There are 52 rows x 118 columns for 6,136 sensing points. The ForeSite PT™ system’s software continuously samples interface pressure at a rate of 1 Hz, which can then be extracted for analysis (Fig. 2). The XSENSOR Pressure Exposure Analyzer Tool (PEAT) will be used to process sessions of pressure readings collected by the ForeSite PT™ system. The new data file produced by PEAT is in a proprietary binary format, and can be exported to TXT or CSV formats for import into a spreadsheet program for analysis (Fig. 3). This will be achieved by the sampling of pressure readings at hourly intervals regardless of whether there has been turning/repositioning when the ForeSite PT™ system’s LCD monitor is turned on and when it is turned off.

#### Treatment group

Inpatients assigned to the treatment group will have the ForeSite PT™ system’s LCD monitor turned on (that is, real-time images of interface pressure will be displayed on the monitor) during their enrollment in the trial.

### Control group

Inpatients assigned to the control group will have the ForeSite PT™ system’s LCD monitor turned off and hidden so that it cannot be accessed by healthcare providers wanting to see pressure readings (that is, real-time images of interface pressure will not be displayed on the monitor). As the ForeSite PT™ system will continue to record interface pressure with the display turned off, this enables patients enrolled in the control group to undergo silent monitoring.

### Outcomes

#### Primary outcome

The primary outcome is as indicated:

1. A composite of interface pressure analysis that reflects the distribution of interface pressure at predetermined time intervals.

These measures include:

- Peak pressure of any given pressure-reading sample.
- Absolute number of sensels with pressure readings greater than 40 mmHg.
- Average interface pressure (excluding sensels with 0 mmHg reading).
- Proportion of patients that have pressure readings greater than 40 mmHg.

Definitions of these measures can be found in Table 1. Given the paucity of literature, this composite measure allows for a greater understanding of the relationship between interface pressure and repositioning strategies. While pressure ulcer incidence may be of primary clinical interest, interface pressure measurements are quantifiable and are recognized to be a major risk factor for pressure ulcer development. For the purposes of this trial, we would like to focus on interface pressure as the primary outcome, as using pressure ulcer incidence as a primary outcome would likely involve many other factors that would influence the development of pressure ulcers, requiring a much higher sample size than what this single-site study can provide.

**Table 1:** Definitions of summary measures of interest

Measure of interest	Description/definition
Mean	Σp_i_/n - average of pressure readings
Peak	P_max_ - maximum pressure reading
Sum	Σp_i_ - sum of all pressure readings
Count ≥40 mmHg	Count of all pressure readings ≥40 mmHg
Standard deviation for average of averages	Headrick, T. C. (2010). *Statistical Simulation: Power Method Polynomials and other Transformations*. Boca Raton, FL: Chapman & Hall/CRC ${\mathrm{V}}_{\mathrm{A}1}=\frac{{\mathrm{m}}^2{\mathrm{V}}_1+{\mathrm{n}}^2{\mathrm{V}}_2\hbox{-} {\mathrm{n}\mathrm{V}}_1\hbox{-} {\mathrm{n}\mathrm{V}}_2\hbox{-} {\mathrm{m}\mathrm{V}}_1\hbox{-} {\mathrm{m}\mathrm{V}}_2+{\mathrm{m}\mathrm{n}\mathrm{V}}_2+\mathrm{m}\mathrm{n}{\left({\mathrm{M}}_1\hbox{-} {\mathrm{M}}_2\right)}^2}{\left(\mathrm{n}+\mathrm{m}\hbox{-} 1\right)\left(\mathrm{n}+\mathrm{m}\right)}\kern0.5em (5.38)$
Average of averages	weighted avg. = [(no. of M)(AVG-of-M) + (no. of R)(AVG-of-R)]/ (total no. of M and R together)

The pressure threshold of 40 mmHg was selected based on analysis of pilot data (not shown) that showed a pressure reading of 39.88 mmHg was at the 90^th^ percentile of the distribution of the pressure measured by the ForeSite PT™ system. This threshold was also considered relevant as existing research indicated that this it has clinical concordance with interface pressure between 30 and 32 mmHg, a range within which there is compromised capillary flow.

#### Secondary outcomes

Secondary outcomes are as described below:

1. A clinical endpoint that measures pressure-related skin and soft tissue changes
  including:

- Skin discoloration.
- Localized tenderness without skin breakdown.
- Stage I and II (partial thickness) pressure ulcer formation.
- Stage III and IV (full thickness) pressure ulcer formation.
- Unstageable/unclassified: full thickness skin or tissue loss - depth unknown.
- Suspected deep tissue injury - depth unknown.
- Presence of any skin, wound or underlying bone infection (that is, cellulitis, infected ulcers or osteomyelitis).

Endpoints c, d, e and f will be identified according to the NPUAP’s pressure ulcer staging system [34]. This outcome will be measured by a clinical head-to-toe skin assessment for detection of pressure-related skin changes and for overt ulceration by the research nurse. This assessment will be conducted within 24 h of admission and on the third day of enrollment. This information will be noted on a form, which requires the research nurse to circle the skin areas where the patient may have skin changes or a pressure ulcer. They will label the circled area with the numbers 1 to 9 that correspond to predetermined categories of pressure-related skin changes and stages of pressure ulcer development (Table 2).

**Table 2:** National Pressure Ulcer Advisory Panel (NPUAP) categories and staging of pressure ulcers and other skin appearance changes to determine pressure of pressure ulceration and severity and development of other skin condition changes

Numbered Labels	Descriptions
1. Pressure-related blanchable erythema (excluding dermatitis, cellulitis, and trauma)	Intact skin with redness; skin remains blanchable on compression, potentially reversible change
2. Stage I pressure ulcer (nonblanchable erythema)	Intact skin with nonblanchable redness of a localized area, usually over a bony prominence. Darkly pigmented skin may not have visible blanching; its color may differ from the surrounding area. The area may be painful, firm, soft, warmer or cooler as compared to adjacent tissue. Category I may be difficult to detect in individuals with dark skin tones. May indicate “at risk” persons.
3. Stage II pressure ulcer (partial thickness skin loss)	Partial thickness loss of dermis presenting as a shallow open ulcer with a red pink wound bed, without slough. May also present as an intact or open/ruptured serum-filled or sero-sanginous filled blister. Presents as a shiny or dry shallow ulcer without slough or bruising*. This category should not be used to describe skin tears, tape burns, incontinence associated dermatitis, maceration or excoriation. *Bruising indicates deep tissue injury.
4. Stage III pressure ulcer (full thickness skin loss)	Full thickness tissue loss. Subcutaneous fat may be visible but bone, tendon and muscle are *not* exposed. Slough may be present but does not obscure the depth of tissue loss. *May* include undermining and tunneling. The depth of a Category/Stage III pressure ulcer varies by anatomical location. The bridge of the nose, ear, occiput and malleolus do not have (adipose) subcutaneous tissue and Category/Stage III ulcers can be shallow. In contrast, areas of significant adiposity can develop extremely deep Category/Stage III pressure ulcers. Bone/tendon is not visible or directly palpable.
5. Stage IV pressure ulcer (full thickness tissue loss)	Full thickness tissue loss with exposed bone, tendon or muscle. Slough or eschar may be present. Often includes undermining and tunneling. The depth of a Category/Stage IV pressure ulcer varies by anatomical location. The bridge of the nose, ear, occiput and malleolus do not have (adipose) subcutaneous tissue and these ulcers can be shallow. Category/Stage IV ulcers can extend into muscle and/or supporting structures (for example, fascia, tendon or joint capsule) making osteomyelitis or osteitis likely to occur. Exposed bone/muscle is visible or directly palpable.
6. Unstageable/Unclassified: Full thickness skin or tissue loss - depth unknown	Full thickness tissue loss in which actual depth of the ulcer is completely obscured by slough (yellow, tan, gray, green or brown) and/or eschar (tan, brown or black) in the wound bed. Until enough slough and/or eschar are removed to expose the base of the wound, the true depth cannot be determined; but it will be either a Category/Stage III or IV. Stable (dry, adherent, intact without erythema or fluctuance) eschar on the heels serves as “the body’s natural (biological) cover” and should not be removed.
7. Suspected deep tissue injury - depth unknown	Purple or maroon localized area of discolored intact skin or blood-filled blister due to damage of underlying soft tissue from pressure and/or shear. The area may be preceded by tissue that is painful, firm, mushy, boggy, warmer or cooler as compared to adjacent tissue. Deep tissue injury may be difficult to detect in individuals with dark skin tones. Evolution may include a thin blister over a dark wound bed. The wound may further evolve and become covered by thin eschar. Evolution may be rapid exposing additional layers of tissue even with optimal treatment.
8. Infection - cellulitis around pressure ulcer	This presents as redness, warmth and swelling in the skin around the pressure ulcers.
9. Infection - pressure ulcer wound base infection, osteomyelitis	This presents as drainage (potentially purulent) with strong odor from the base of the pressure ulcer. May have necrotic material as wound base.

1. Perceptions (both positive and negative) of healthcare providers caring for patients allocated to the treatment group to the CPI system. This outcome will be measured by a survey consisting of 17 close-ended and open-ended questions about prior experience with pressure mapping technology, functionality, ease of use, and interpretation of pressure data on the LCD monitor. These questions were utilized in a prior pilot study assessing healthcare providers’ perceptions to the interface pressure information provided on the LCD monitor, and the impact of this information on patient care. The survey will be administered to healthcare providers approximately three months after enrollment has begun on their respective units, and will take approximately ten minutes to complete.
2. Perceptions (both positive and negative) of patients (and if appropriate, family members) allocated to the treatment group to the CPI system. This outcome will be measured by a survey consisting of seven close-ended and open-ended questions about prior and current experience with CPI (including sensor mattress cover and monitor display of their pressure distribution) on their care and comfort. The survey will be administered by the research nurse or the research assistant on the patient’s third day of enrollment and will take approximately 10 minutes to complete.

#### Additional data collection

To establish a patient profile at baseline (within 24 h of admission), a pressure ulcer risk assessment with the Braden Scale will be conducted, and demographic and medical history information will be collected from patient charts. Table 3 specifies the information that will be collected at baseline, on the second day of enrollment and on the third day and/or last day of enrollment (depending on whether the participant is in the main sample or the subsample). The risk assessment will be conducted by the research nurse, and demographic and medical history information will be collected by either the research nurse or the research assistant.

**Table 3:** Schedule of data collection

Data to be collected	Day 1: Baseline (within 24 hours of admission)	Day 2: Interim	Day 3: Enrollment termination (within 24 hours)*
Continuous interface pressure	Yes	Yes	Yes
Clinical head-to-toe skin assessment	Yes	No	Yes
Braden Scale	Yes	No	Yes
Level of bed mobility	Yes	No	Yes
Charlson comorbidity index	Yes	No	No
Complete blood count (CBC)	Yes	No	No
Pre-albumin	Yes	No	No
Albumin	Yes	No	No
Creatinine	Yes	No	No
Liver function test results (alkaline phosphatase; aspartate aminotransferase (AST))	Yes	No	No
C reactive protein (CRP)	Yes	No	No
Glycosylated hemoglobin	Yes	No	No
Body mass index (BMI)	Yes	No	No
Reason for admission/diagnoses	Yes	No	No
Bladder management	Yes	No	No
Bowel management	Yes	No	No
History of cancer and treatment	Yes	No	No
History of cardiovascular disease	Yes	No	No
History of renal failure	Yes	No	No
Use of immunosuppressive medications	Yes	No	No
Pressure ulcer history	Yes	No	No
Smoking history	Yes	No	No
Illicit drug use	Yes	No	No
Demographics (that is, sex, date of birth, race)	Yes	No	No
Perceptions of healthcare providers of patients in the treatment group**	No	No	No
Perceptions of patients (and/or their family members) in the treatment group	No	No	Yes
Pressure ulcer prevention and treatment methods	No	No	Yes

*For the subset sample of 60 participants, this information will be collected on the day of discharge from the hospital, as well as on weekly basis if their length of stay exceeds one week

**This information will be collected approximately 3 months after enrollment has begun on their respective units to ensure healthcare providers have sufficient exposure to the technology

The Braden Scale [26], which was developed to assist healthcare providers with assessing a patient’s level of risk for pressure ulcer development and inform the course of a particular treatment, will be administered to each patient to determine their individual risk. It is a summated rating scale composed of six subscales: sensory perception, activity, mobility, moisture, friction and nutrition. Each subscale is scored from 1 to 3 or 1 to 4, for a total score that ranges from 6 to 23. A lower total score indicates a lower level of functioning and therefore a higher level of risk for pressure ulceration.

In addition, on the third and last day of enrollment, the research nurse or research assistant will observe and note the pressure ulcer prevention and management strategies that were used (for example, wedges) for each participant.

### Sample size

A total of 678 patients will be recruited from Units 36, 37, 101, 112 and the ICU for enrollment in the trial, in order to randomize 339 patients to each of the intervention and control arms. Sample size estimates for the primary outcome indicate a need for 308 patients in each of the intervention and control arms, in addition to 31 patients per arm to allow for 10 % attrition. This is based on an analysis of pilot data (not shown) that showed a mean pressure of 136 mmHg (SD = 62 mmHg) and allows for the detection of a statistically significant 15 % relative decrease in the count of sensels over 40 mmHg (β 0.2 that is, Type II error rate).

The subsample of 60 participants will be purposively sampled from the same units to ensure that a wide range of length of stays will be represented. Because the objective of extracting a subsample of participants is to inform a potential larger multicenter trial rather than to sufficiently power a substudy, 60 participants were deemed to be sufficient to provide a longer term snapshot of patients who have lengths of stay longer than 3 days.

### Randomization

A randomized treatment allocation table was created on 24 November 2014, using sealedenvelope.com [35]. The seed was 57107341298070; the treatment groups were: Screen ON and Screen OFF, Block Sizes were 4, 6 and 8; the length of the list was 5024, and were stratified by unit: ICU, Unit 101, Unit 111, Unit 112, Unit 36, Unit 37.

Study data will be collected and managed using REDCap [36] electronic data capture tools hosted at the University of Calgary in collaboration with the Cumming School of Medicine. The randomization module was enabled in REDCap, and the allocation table produced by Sealed Envelope was imported into the web-based application. Patients will be entered into the database and randomized according to their care unit.

### Blinding

Allocation will be independently undertaken, and concealed from principal investigator, co-investigators and the research coordinator, who will be involved in data analysis. The research nurse and the research assistant will both be involved in patient recruitment, the randomization process and data collection, and will not be blinded to treatment allocations. Outcome assessment for clinical data will not be blinded as the outcome is objective, and it has been found that there is little evidence of bias in trials with objective outcomes [37]. It will not be possible to blind patients and healthcare providers because they will be able to see if the LCD monitor that displays information about the patient’s surface pressure on the bed is mounted to the headwall.

### Analysis and statistical methods

Data analysis will use univariate statistics to summarize changes in pressure distribution as per the interface pressure feedback collected by the CPI system. Chi-square and/or Fisher exact tests will be used for between-group (that is, intervention and control groups) comparisons of the primary outcome and the secondary outcomes, and the Wilcoxon rank sum test for between-group comparison of strategic shifting. Chi square and t-tests will be used to compare baseline characteristics between groups, and both linear least squares regression and logistic regression will be used for adjusted analyses if there are notable between-group differences for important baseline characteristics. Correlation between pressure distribution (the study’s primary endpoint) with the hard clinical outcome (pressure ulceration and pressure-related skin appearance changes) will also be explored.

Simple descriptive statistics will be used to summarize close-ended survey data, whereas open-ended survey data will be thematically analyzed and reported qualitatively.

Missing data will be dealt with by analyzing data using an as-treated approach. A sensitivity analysis will explore the results as per protocol (all patients regardless of missing data) to determine bias that might exist. Depending on the amount of missing data, imputing missing values may be performed and models compared.

## Discussion

While it is generally accepted that prolonged interface pressure contributes to the development of ulcers, limited research exists in this area, particularly on the role of CPI. This is the first randomized controlled trial to assess the efficacy of CPI for reducing interface pressure and on the development of pressure-related skin and soft tissue changes. This RCT will compare the interface pressure and pressure-related skin and soft tissue changes between the intervention group, which will have the ForeSite PT™ system with the LCD monitor turned on to provide visual feedback through CPI to the clinicians, and the control group, which will have the ForeSite PT™ system with the LCD monitor turned off, therefore not providing visual feedback or CPI to the clinicians. Both groups will undergo continuous interface pressure data collection and clinical assessment. Thus, the present RCT will contribute to a better understanding of the role of CPI in interface pressure management and, subsequently, the prevention and management of pressure ulcers.

While this RCT in itself is not likely to be sufficient to inform health system decision making on whether to purchase CPI systems en masse, the findings could provide greater understanding of the use of this technology in clinical practice. This study also has the potential to increase our understanding of the relation between interface pressure and skin and tissue changes resulting from the exposure to prolonged pressure. A much larger trial would be required in subsequent stages to determine the impact of CPI on the hard clinical endpoint of overt pressure ulceration, that is, the clinical outcome that we are ultimately trying to prevent. The present RCT described here will build significantly on previous research on interface pressure and pressure ulcer development, and it will clarify the role of CPI in modifying interface pressures, an important intermediate goal in the prevision pressure ulcers.

## Trial status

To date, a total of 117 participants have been recruited; of these, 105 have been included in the trial, and 12 were excluded.

## Abbreviations

AIHS: Alberta Innovates Health Solutions
BMI: body mass index
CBC: complete blood count
CPI: continuous pressure imaging
CRIO: Collaborative Research and Innovation Opportunities
CRP: C reactive protein
ForeSite PT™ system: ForeSite PT™ Patient Turn System
ICU: intensive care unit
LCD: liquid crystal display
RCT: randomized controlled trial
